# Robotically Assisted Gynecologic Surgery: 2-Year Experience in the French Foch Hospital

**DOI:** 10.3389/fsurg.2014.00008

**Published:** 2014-05-05

**Authors:** Julie Goetgheluck, Marie Carbonnel, Jean Marc Ayoubi

**Affiliations:** ^1^Service de gynécologie-obstétrique, Hôpital Foch, Suresnes, France

**Keywords:** robotic laparoscopy, hysterectomy, myomectomy, adnexal surgery, sacrocolpopexy

## Abstract

Robotically assisted laparoscopic surgery has seen rapid expansion over the past few years and it constantly evolves with a progressive enlargement of its range of indications. In the present paper we would like to share our 2-year experience regarding the use of robotics in various laparoscopic procedures, including hysterectomy, myomectomy, adnexal surgery, and sacrocolpopexy.

## Introduction

Robotic surgery is one of the leading advances in the evolution of minimally invasive surgery over the past decade.

In gynecologic surgery, robotically assisted laparoscopy has seen rapid expansion, mainly since 2005 when the use of the Da Vinci^®^ system has been agreed in the United States for hysterectomy procedures (1). This technological advance offers numerous advantages over conventional laparoscopy. Surgeon’s gestures gain accuracy owing to the robot ability to filter and reduce physiologic tremor. This device has also the ability to transform surgeon’s hand movements into more precise micro-movements of the jointed-wrist instruments that operate with seven degrees of freedom and 360° pronosupination amplitude. An improved 3D HD vision provided by a camera directly controlled by the operator enables accessing deep area location. Sutures gain quality as a result of both continuity and stability of the hand movements, making gesture intuitive. Moreover, the ergonomic quality of the console enhances comfort and stability.

Compared with laparotomy and probably laparoscopy, robotic technology appears to reduce blood loss, postoperative pain, morbidity, and hospital-related cost and duration of stay (2–4).

Yet, the use of robotic surgery remains less than expected, mainly due to both the cost of the device and its maintenance, and the prolonged duration of occupation of the operative room. Two other limitations are the lack of haptic feedback and the long training period of the surgeons (5), although learning curves seem to show reduced time of training compared with conventional laparoscopy.

This paper reports all cases of robotically assisted gynecologic procedures carried out in our department of gynecology between 2010 and 2012, mainly hysterectomies for benign or malignant disease, myomectomies, adnexal surgery, and surgical procedures for pelvic floor disorders.

## The Place of Robotics in Gynecologic Surgery: 2-Years of Experience in Foch Hospital and Literature Data

Our series includes all patients having undergone robotically assisted laparoscopy for benign or malignant disease between January 2010 (date of robot acquisition in the department) and January 2012. Two surgeons were involved in the procedures. The Da Vinci^®^ SI System was used for all cases. The operations were performed after patients have provided their informed consent.

The procedures were total hysterectomies (conservative or not), myomectomies, distal adnexal surgery and proximal tubal reanastomosis, and prolapse surgery (sacrocolpopexy).

For all interventions, we recorded the patients’ characteristics: age, BMI, parity, surgical history, hormonal status, fertility. Procedures were also described in terms of operating time, anesthesia duration, blood loss, intraoperative complications, number of laparoconversions, and postoperative data (duration of hospital stay, postoperative complications).

In the present paper, we assess our results compared to the literature data (Table 1).

**Table 1:** **Results**.

Surgery	Number	Mean operating time (min)	Mean anesthesia time (min)	Mean blood loss (ml)	Laparoconversion	Peroperative complications	Postoperative complications	Length of hospital stay (day)
Hysterectomy	75	128 (60–250)	192 (105–306)	58 (0–650)	2	2	3	3.5 (2–8)
Myomectomy	18	149 (65–282)	213 (115–375)	320 (50–600)	0	0	2	2.3 (1–11)
Tubal reanastomosis	4	200	230	20 (10–50)	0	0	0	2 (1–4)
Sacrocolpopexy	5	260 (160–290)	305 (180–330)	60 (10–100)	0	0	0	2 (1–3)

*Min, minutes; (–), smallest and largest value; ml, milliliter*.

### Preparation of the procedure and methods for robot installation

Irrespective of the nature of the procedure, the patient was hospitalized 1 day before the intervention. Bowel preparation was carried out in order to improve visibility and intestinal mobilization.

The patient was installed in the operating room in the lithotomy position. Measures were taken to prevent potential falls, such as the use of silicone gel and shoulders blocking. The patient’s arms were protected, immobilized, and fixed alongside the body. Maximal Trendelenburg position was tested prior to draping in order to control patient adequate immobilization and cardiopulmonary tolerance.

Following gas insufflation, laparoscopic trocars placement, and docking of the three or four arms of the robot, the surgeon operated from the console. The operating assistant remained beside the patient in order to perform aspiration or exposure with the assistant port.

Docking was performed with the robotic cart positioned laterally next to the patient, oriented 45° toward the contralateral shoulder to allow lower surgical access. The optical trocar was always placed above the upper umbilical area in order to widen the field of view. The two other 8 mm trocars were positioned laterally at a distance of 10–20 cm on both sides of the optical port. The assistant port was positioned 5 cm above the right lateral iliac spine.

### Hysterectomies

For such procedures, we used the Clermont-Ferrand uterine device that allows optimal exposure of the uterus and a very safe realization of the colpotomy. We operated using monopolar scissors in the right hand and the windowed bipolar grasping device on the left. Once the uterus was exteriorized through the vagina, vaginal sutures were performed using two needle-holders allowing x-shape Vicryl 1/0^®^ stitches.

We carried out 75 hysterectomies. Patient’s mean age was 45 years (21–84), and mean BMI was 25.15 kg/m^2^ (18–37). In this sample, 40.25% were multipara (*n* = 31), 26% had a history of laparotomy (*n* = 19), and 26% were menopausal women (*n* = 19).

The indications for hysterectomy for benign disease were primarily metrorrhagia or pelvic pain related to fibroid uterus in 35.5% (*n* = 27), Benjamin syndrome in 29% (*n* = 22), endometrial hyperplasia with or without atypia in 21.2% (*n* = 16), and adenomyosis in 5.3% (*n* = 4). Four hysterectomies were carried out for intraepithelial cervical neoplasia and three for stage I endometrial cancer.

Mean operating time was 128 min (60–250), mean console time was 93 min (30–180), mean anesthesia duration was 192 min (105–360), and mean blood loss was 58 ml (0–650). Mean weight of uterus was 160 g (29–656). Mean duration of hospital stay was 3.5 days (2–8).

Two laparoconversions occurred (2.6%): the first was due to non-removable enlarged uterus and the second was secondary to pelvic adhesions. Two intraoperative complications were reported: one serous bowel injury and one bladder injury; both were immediately sutured (one Vicryl 3/0^®^ stitch for bowel injury, and two plans of vicryl for bladder injury). Three postoperative complications occurred among the 75 procedures (3.9%): one pelvic abscess treated by antibiotherapy, one collection in Douglas pouch, and one infection of the fascia.

Robotically assisted laparoscopic hysterectomy has been evaluated by numerous authors.

Payne and Dauterive (2) published a retrospective study comparing 100 cases of robotically assisted laparoscopic hysterectomy vs. 100 conventional laparoscopy cases; this study reports similar uterine weights, operating times, and blood losses in the two types of procedures. Mean operating time was 119 min with the robot along with a mean uterine weight of 267 g. More laparoconversions occurred with conventional laparoscopy: 5.9 vs. 4% with the robot and, in the last 25 cases of robotic hysterectomy arm there was a shorter operating time compared to conventional procedures (2).

Landeen et al. (5) carried out a retrospective study of 1474 hysterectomies, comparing four techniques: abdominal, vaginal, conventional, and robotically assisted laparoscopy. Their analyses show reduced blood loss and hospital stay with robotic surgery (*p* < 0.0001), and higher overall complication rate with the laparotomy (14%); this rate being the lowest with the vaginal technique; the conversion rate is fourfold higher with the laparoscopic technique (5).

Robotically assisted laparoscopy is associated with the shorter duration of hospital stay: 1.35 days (91 patients) according to Kho et al. and 1 day (100 patients) for Lenihan et al. (6, 7).

Complication rates were also reported: 2% for Payne et al., 3.5% for Lenihan et al., and even 6% for Kho et al. (2, 6, 7).

Our results are in accordance with those reported in the literature regarding the duration of operating time, uterine weight, and blood loss with the robotic technique (2, 6–9), but our complication rate is lower. We observed some progressive improvement after 10–20 cases of our series regarding operating times and conversion rate although the procedures were more difficult, with enlarged multifibroid uterus.

### Myomectomies

Myomectomies were done for interstitial and sub-serosal fibromas either in the case of infertility or in the case of symptomatic fibromas (pelvic pain and metrorrhagia). Robotically assisted laparoscopic myomectomy is performed using the monopolar scissors and dipolar grasping device with sometimes the use of the fourth arm of the robot for myoma traction. The myomectomy area is sutured as two myometrial planes by x-shaped Vicryl 0^®^ stitches and the uterine serosa is sutured by monocryl 4/0^®^ overedge stitches in order to reduce the risk of adherence. Finally, we extract the myoma using the assistant port and the Storz^®^ Rotogut G1 morcellator after withdrawing the robot arms for more safety.

Eighteen patients underwent robotically assisted laparoscopic myomectomy, of whom three had fertility disorders. Their mean age was 37.9 years (27–46) and their mean BMI was 28 kg/m^2^. One patient out of 4 had a history of abdominal-pelvic surgery; 3 patients had never been pregnant, 10 were primiparous and 5 were multiparous women.

In our series there were various indications for myomectomy: infertility, pelvic pain, heaviness, and metrorrhagia.

Mean operating time was 149.25 min (65–282), mean anesthesia time was 213 min (115–375), mean fibroma weight was 149.7 g (49–332), and mean fibroma size was 7.7 cm (5–15). In 13 cases, 1 myoma was removed, in 4 cases 2, and in 1 case 3. These interventions did not necessitate opening the uterine cavity. Mean blood loss was estimated to be 320 ml (50–600).

No laparoconversion occurred. Postoperative complications were a digestive hemorrhage in one patient after gastric ulcer perforation (history of treated ulcer) 4 days post-surgery, and one patient experienced wall hematoma.

Several comparative studies on this topic have been published.

Evaluation of robotic myomectomies has not shown any specific morbidity (10). Advincula et al. (11) have published a retrospective study comparing conventional laparotomy with robotic laparoscopy. In this study, the robotic surgery needed longer operating time: 231.38 ± 85.1 vs. 154.41 ± 43.14 min (*p* < 0.05) but with this technique blood loss and duration of hospital stay were significantly shorter; laparotomy was associated with higher rates of morbidity (11).

The longer operating time associated with the robotic technique is related to the time necessary to install the robot and to myoma extraction by morcellation. The time for installation decreases with experience acquisition (7). It should be noticed that no case of laparoconversion occurred in our series, whereas in the literature, rates of 8–15% were reported (12).

In the context of infertility management, myomectomy improves pregnancy rates by spontaneous conception for intra-mural fibromas >5–7 cm (13). In patients undergoing medically assisted reproduction, intra-mural myomas with or without intra-cavitary development have a deleterious effect on fertility parameters, and pregnancy rates decrease when the myoma is more than 4 cm (13).

In 1931, Bonney defined abdominal myomectomy as the preferential treatment to women wishing to preserve their fertility (14). In 1979, Semm demonstrated the feasibility of laparoscopic myomectomy (15). Several authors underline the difficulty of laparoscopic suturing (12, 16, 17) and the risk of secondary uterine rupture remains unknown. Robotically assisted laparoscopic myomectomy may improve the quality of the sutures.

The results of those studies that compared robotic myomectomy vs. conventional laparoscopic myomectomy showed reduced blood loss and hospital stay and conversion rates = 0 when the laparoscopy was robotically assisted (18–20).

In 2010, Ascher-Walsh and Capes (21) have compared 75 robotically assisted laparoscopic myomectomies vs. 50 conventional laparoscopic myomectomies: extended operating time is reported with the robotic technique (192 vs. 138 min) but blood loss is reduced (226 vs. 459 ml) and the duration of hospital stay is shorter (0.5 vs. 3.3 day).

Regarding long-term outcome, larger studies are needed, aimed at evaluating the pregnancy rate after myomectomy and the risk of secondary uterine rupture with the robotically assisted laparoscopy vs. laparotomy.

### Adnexal surgery

Eleven patients underwent adnexal surgery in our department: four underwent tubal plasty, four underwent proximal tubal reanastomosis after a history of proximal tubal ligation, and three had a salpingectomy rather than tubal plasty due to hydrosalpinx, and worsened tubal prognosis related to the intraoperative presentation of the tubal mucosa.

Tubal reanastomosis following ligation by Filshie coagulation section or clip was performed after resection of the sclerotic area using monopolar scissors and dipolar grasper. Reanastomosis was carried out by five extra-mucosal stitches of Prolene 5/0^®^. No intra tubal guide was used during procedure. A tubal methylene-blue test was performed at the end of the procedure.

In the group of eight women who underwent tubal plasty or reanastomosis, mean age was 36.5 years, 87.5% were multipara, and 12.5% were primipara. Mean operating time was 148 min, and mean anesthesia time was 195 min. Mean operative time for tubal anastomosis was 200 min. 50% of the interventions necessitated adhesiolysis.

At the end of the intervention, the methylene-blue test was bilaterally positive in 50% of the cases, unilaterally in 25%, and negative in 25%. No intraoperative complication occurred.

Four pregnancies were obtained of which one was ectopic; the test of tubal permeability in this case was unilaterally positive. Two full-term pregnancies occurred 3 months post-surgery and the ectopic pregnancy occurred 9 months after the intervention.

The first case of robotically assisted tubal anastomosis has been described by Falcone et al. in 1999 (22). Dharia Patel et al. (23) retrospectively compared robotically assisted laparoscopic tubal anastomosis performed in 18 patients vs. laparotomy in 10; the same operator was involved and no other factor of infertility existed. In the robotic surgery group, compared with laparotomy the operating time was longer (201 vs. 155 min) but the console time was 156 min. Both duration of hospital stay and time to return to normal active life (11 vs. 28 day) were significantly shorter with the robotic technique. In this group the pregnancy rate after 1 year was 62.5 vs. 50% in the group having undergone laparotomy (ns). Similar results were reported in 23 patients by Rodger et al. in 2007 (24).

Despite the existence of biases in these studies, these results and those of our series suggest numerous advantages of robotic-assisted laparoscopy compared with laparotomy regarding postoperative outcome and pregnancy rate, owing to the techniques of microsurgery.

Goldberg and Falcone (25) carried out a retrospective study that compared the robotic system “Zeus” (10 patients) with laparoscopy (15 patients); robotic surgery was associated with longer operating time, more blood loss, and a trend for higher pregnancy rate (50 vs. 37.5%, ns). The retrospective study of Caillet et al. (26) observed a pregnancy rate of 71% 24 months post-surgery in 97 patients who underwent robotically assisted surgery.

Other studies are needed however to determine the advantage of robotically assisted tubal reanastomosis regarding costs, operating time, and pregnancy rates.

### Sacrocolpopexy

Anterior and posterior polyester type III Mersuture^®^ prostheses were fitted and fixed using only Tycron 2/0^®^ stitches. We used also an intravaginal malleable valve and an intrarectal dilator to improve exposure.

Five patients had their prolapse cured by sacrocolpopexy and we performed a sacrocolpopexy prosthesis resection in a sixth one. No uterus was removed during the procedure. Prosthesis resection was necessary for one patient having undergone laparoscopic sacrocolpopexy with another surgical team few years ago. Prosthetic vaginal erosion was observed in this patient. Mean operating time for sacrocolpopexy was 260 min (160–290), mean anesthesia time was 305 min (180–330); no intraoperative complication occurred and mean hospital stay was 2 days. At the end of the interventions, we observed at clinical examination complete correction of pelvic floor statics. No complications were observed at months 1 and 3 postoperative visits.

Robotic assistance appears to be particularly suitable to laparoscopic sacrocolpopexy since refined dissection of the vesico-vaginal, recto-vaginal, and presacral areas is necessary, as well as for the suturing process. We do believe that the aid provided by the robot facilitates the procedure and reduces the operating time necessary for suturing prosthesis on the vagina, elevator muscles, and sacrum.

One of the larger series that evaluated this technique is that of Geller et al. (27); this study compares a series of 73 robotically assisted laparoscopic interventions operated by the same surgeon with another series of 105 laparotomic procedures performed by another surgeon. Patients were comparable regarding their age, BMI, and history of surgical interventions. However, the patients in the robotic surgery group had a more important prolapse as assessed by the preoperative POP-Q score and more associated indication for hysterectomy. Operating time was longer with the robotic technique but less blood loss and shorter hospital stay were reported. Equivalent complication rate was reported for the two groups and the postoperative evaluation at 6 months regarding the prolapse treatment showed identical results (27, 28).

A study evaluated the long-term postoperative results in 21 patients after a mean follow-up of 24 months: the authors reported a rectocele recurrence in 1 patient, 1 recurrent vaginal prolapse, and 2 prosthesis erosions (29).

Further randomized studies remain necessary to determine whether robotic laparoscopic sacrocolpopexy is superior to other techniques regarding complications, return to normal life, and above all functional outcome.

## Conclusion

Our 2-year experience in robotically assisted gynecologic surgery allows us to highlight interesting advantages of this technique, and enlarge the range of its operative indications. In fact, the use of the da Vinci^®^ system helped us observe potential benefits for our patients despite the fact that the lack of control group in our study is a major limitation. In the literature, in comparison to laparotomy and even to conventional laparoscopy, this technique is likely to reduce blood loss, allow earlier return to normal life, and carry less postoperative complications especially after complex surgery. Robotically assisted surgery has been used in our institution in numerous surgical gynecologic indications for benign disease: prolapse, endometriosis, myomectomy, adnexal surgery, etc. Irrespective of the nature of the operation, the technical feasibility of the procedure was demonstrated.

Further randomized studies are needed to significantly determine the superiority of robotic surgery over other techniques so as to implement it more easily in gynecological procedures. However, cost and affordability remain today as serious limitations to the expansion of robotically assisted surgery (30).

## Conflict of Interest Statement

The authors declare that the research was conducted in the absence of any commercial or financial relationships that could be construed as a potential conflict of interest.
